# Decision-making support systems on extended hospital length of stay: Validation and recalibration of a model for patients with AMI

**DOI:** 10.3389/fmed.2023.907310

**Published:** 2023-02-08

**Authors:** Joana Xavier, Joana Seringa, Fausto José Pinto, Teresa Magalhães

**Affiliations:** ^1^NOVA National School of Public Health, Nova University Lisbon, Lisbon, Portugal; ^2^Serviço de Cardiologia do Centro Hospitalar de Lisboa Norte, Faculdade de Medicina, Universidade de Lisboa, Lisbon, Portugal; ^3^Public Health Research Centre, Comprehensive Health Research Center, CHRC, Nova University Lisbon, Lisbon, Portugal

**Keywords:** acute myocardial infarction, cardiovascular diseases, length of stay, predictive models, decision-making

## Abstract

**Background:**

Cardiovascular diseases are still a significant cause of death and hospitalization. In 2019, circulatory diseases were responsible for 29.9% of deaths in Portugal. These diseases have a significant impact on the hospital length of stay. Length of stay predictive models is an efficient way to aid decision-making in health. This study aimed to validate a predictive model on the extended length of stay in patients with acute myocardial infarction at the time of admission.

**Methods:**

An analysis was conducted to test and recalibrate a previously developed model in the prediction of prolonged length of stay, for a new set of population. The study was conducted based on administrative and laboratory data of patients admitted for acute myocardial infarction events from a public hospital in Portugal from 2013 to 2015.

**Results:**

Comparable performance measures were observed upon the validation and recalibration of the predictive model of extended length of stay. Comorbidities such as shock, diabetes with complications, dysrhythmia, pulmonary edema, and respiratory infections were the common variables found between the previous model and the validated and recalibrated model for acute myocardial infarction.

**Conclusion:**

Predictive models for the extended length of stay can be applied in clinical practice since they are recalibrated and modeled to the relevant population characteristics.

## Introduction

Europe has been undergoing profound demographic and social changes, the most visible of which are increases in average life expectancy and the increasing number of elderly people (1). The aging of the population, associated with the increase in chronic and degenerative diseases, is a phenomenon that represents a significant economic, health and social challenge for healthcare systems (2).

Among chronic diseases, cardiovascular diseases have emerged as the leading cause of death globally, representing 32.0% of all global deaths in 2019 (3).

In Portugal, diseases of the circulatory system accounted for 29.9% of total deaths (33,624 deaths), in 2019, an increase of 2.1% from the previous year. There were 10,975 deaths from cerebrovascular diseases, 7,151 deaths from ischemic heart disease and 4,275 deaths caused by acute myocardial infarction (AMI) (4), in the group of causes motivated by circulatory system diseases.

In Portugal, there was an 8.1% decrease in the number of hospitalizations for circulatory system diseases compared to 2011, with this decrease being especially relevant in hospitalizations for AMI, which accounted for 10.4% of hospitalizations for circulatory systems diseases (11,510 episodes) in 2016 (5). This shift may be explained by investments in strategic preventive measures and improved diagnosis in the areas of AMI and stroke (5). However, there was an increase in the total number of days patients were hospitalized for AMI between 2010 (91.060 days) and 2014 (95.315 days) (6).

The length of stay (LOS), which refers to the number of days spent in a hospital by each patient (7), is commonly considered to be a measure of efficiency and a proxy for hospital resource consumption (8). It also provides better understanding of patient flow, which is essential to understanding both the operational and clinical functions of a healthcare system (9–11).

Reducing LOS contributes to lower costs and improved outcomes for patients (12), therefore there is a growing interest in the development of models to predict LOS.

The predictive model for extended length of stay (LOSE) by Magalhães et al. (13), has identified specific variables that lead to an increased risk of LOSE in patients with AMI: comorbidities (diabetes with complications, cerebrovascular disease, shock, respiratory infections, pulmonary edema, cardiac dysrhythmia), altered partial pressure of oxygen in the blood (pO2), being aged 69 years or older, and with neutrophils and prothrombin time above level.

Administrative data, which are readily available, relatively inexpensive to obtain, easily accessible, and widely used to assess resource use of hospital systems (14–16), can be used to build the LOS predictive model. The use of administrative data has limitations, such as inaccurate data, coding errors, missing cases, and other inconsistencies; however, it is commonly the only source of information available to observe and analyze clinical issues. It should also be noted that these data could be used to identify quality indicators and benchmark hospital activity (14, 16).

As the prevalence and incidence of cardiovascular diseases in Portugal are significant, as is their weight in hospital management, both in terms of costs, bed occupancy and discharge management, it is imperative and relevant to evaluate the added value of using predictive models in the daily practice of healthcare facilities.

Therefore, the purpose of this study is to determine whether predictive models of LOS at the time of admission developed for small hospital populations can be generalized to larger populations by identifying patients with extended LOS (LOSE).

The specific objectives of the study were to:

- Validate the predictive models for an extended length of stay in a new population with the algorithm previously developed by Magalhães et al. (13), taking into account the set of variables and coefficients determined;
- Recalibrate the model in the new population and verify if the model changes substantially.

## Materials and methods

### Study population

The study population included patients aged 18 years or older, discharged alive, whose primary diagnosis of admission was AMI (410 ICD-9-CM). To identify the episodes, the codes of the International Classification of Diseases, 9th Revision, Clinical Modification (ICD-9-CM) were used. Episodes of AMI coded as subsequent diagnosis were excluded, as well as episodes from patients transferred to another hospital. The final sample included 1,531 episodes.

While the Magalhães et al. (13) study used laboratory and administrative data from a Portuguese NHS hospital (~400 beds) in 2010–11, the study population in the current study differs in terms of the period of analysis, sample size, and the hospital from which the data reports.

### Data collection

This study used administrative and laboratory data discharges from a National Health Service (NHS) large hospital in Portugal (~1,000 beds), from 2013 to 2015. The anonymized database includes demographic data, such as sex and age, type of admission, primary and secondary diagnosis, and medical procedures (ICD-9-CM), destination after discharge, and analytical results. The study was approved by the hospital's ethics commission (reference 19/16). The data obtained were kept anonymous and confidentiality was ensured. To safeguard the identity of the study population, the hospital guaranteed the anonymity of the data, and the database was made available with encrypted identification codes.

### Methods

The study method is 2-fold: first, to validate the prediction of the extended length of stay (LOSE 7 days) on patients with a primary diagnosis of AMI who were discharged alive using the predictive model developed by Magalhães et al. (13), also known in this study as the validated model (VM). Second, the same algorithm used in the Magalhães et al. predictive model was used to recalibrate the model, but the study population focused on patients with LOSE ≥11 days in the sample of patients with the primary diagnosis of AMI. In this study, the recalibration model is referred to as RM.

LOSE was the outcome variable used to promptly identify patients at higher risk of prolonged hospitalization, identifying patients with an adverse result or excessive LOS (value:1). Patients with LOS greater than the 75th percentile (≥7 days in VM and ≥11 days in RM) were considered to have LOSE (5, 16). LOS was defined as the time in days between admission and discharge to the inpatient hospital setting. The different LOSE in the two models corresponds to the use of the same LOSE ≥7 days in the VM (anterior sample model) and the use of the corresponding LOSE on the actual sample ≥11 days in RM.

The model variables determined by Magalhães et al. (13) were: diabetes with complications, cerebrovascular disease, shock, respiratory infections, pulmonary edema, pO2 above level, age group of 69 to 100 years old, cardiac dysrhythmia, neutrophils above level, and the duration of prothrombin above level.

The missing values on laboratory data were handled by applying normal levels, thus using a single imputation procedure (13).

### Statistical analysis

A multiple logistic regression based on the previously developed model was used to estimate the coefficients of each variable, and the odds ratio (OR) was used to analyze the coefficients of each variable for VM.

In the RM, a combination of all the independent variables recorded in the population's database under study was used: laboratory results, sex, age, type of AMI and comorbidities.

The final algorithm was created by using the same method as the training model developed by Magalhães et al. (13), so all the data was used to recalibrate the model. Therefore, a simple logistic regression was used to select variables for the multiple analysis. The Wald test was used in this analysis, with a significance level of 25% (*p-*value < 25%) considered for this phase.

The variable selection methods used for the multiple analysis were stepwise forward and stepwise backward, that presented the best Akaike Information Criterion (AIC) as a reference.

Odds ratios were used to analyze the model coefficients.

The predictive capacity of the models was assessed using the following parameters: predictive efficiency, discriminatory capacity, and calibration.

## Results

### Descriptive statistics study population

As shown in Table 1, male patients account for 66.4% of 1,531 episodes of patients discharged alive whose primary diagnosis was AMI, while female patients account for 33.6%. 48.6% of the episodes had a LOSE ≥7 days and 26.5% a LOSE ≥11 days.

**Table 1:** Descriptive statistics of the study population—Administrative data.

	**Discharged alive with principal diagnosis of AMI**		**LOSE** ≥**7 days**		**LOSE** ≥**11 days**	
	***N***	**%**	***N***	**%**	***N***	**%**
Total	1,531	100	744	48.6	406	26.5
**Sex**						
Male	1,016	66.4	470	46.3	270	26.6
Female	515	33.6	274	53.2	136	26.4
**Type of AMI**						
Anterior STEMI	256	16.7	121	47.3	62	24.2
Other STEMI	662	43.2	319	48.2	164	24.8
NSTEMI	613	40.0	304	49.6	180	29.4
**Comorbidities**						
Anemia	191	12.5	135	70.7	94	49.2
Cancer	63	4.1	29	46.0	16	25.4
Cardiogenic shock	31	2.0	31	100	28	90.3
Diabetes with complications	129	8.4	86	66.7	60	46.6
Diabetes without complications	342	22.3	186	54.4	95	27.8
Cardiac dysrhythmia	379	24.8	232	61.2	141	37.2
Cerebrovascular disease	173	11.3	110	63.6	61	35.3
Pulmonary edema	68	4.4	61	89.7	48	70.6
Respiratory infection	164	10.7	144	87.8	99	60.4
Acute kidney failure	294	19.2	184	62.6	116	39.5
Chronic kidney failure	302	19.7	191	63.2	120	39.7

Of the episodes of patients with LOSE, the AMI NSTEMI-type (29.4%) presented the highest LOS. Of the 31 episodes with shock comorbidity, 28 (90.3%) had an LOSE, followed by patients with pulmonary edema (70.6%) and respiratory infection (60.4%).

Regarding laboratory data, as shown in Table 2, most patients with AMI discharged alive have abnormal levels of lymphocytes (80.6% above level), neutrophils (81.8% above level) and troponin I (86.2% above level). Almost all the results above level are in the episodes with LOSE ≥7 days.

**Table 2:** Descriptive statistics of the study population—Laboratory data.

**Variable**	**Below level**				**Above level**			
	***N***	**%**	**LOSE** ≥**7 days (%)**	**LOSE** ≥**11 days (%)**	***N***	**%**	**LOSE** ≥**7 days (%)**	**LOSE** ≥**11 days (%)**
Albumin	88	5.75	72.7	59.09	3	0.20	66.7	0
Calcium	170	11.10	56.5	38.24	6	0.39	50.0	33.33
Chlorine	51	3.33	58.8	37.25	179	11.69	39.7	19.55
Creatine kinase (CK)	9	0.59	66.7	55.56	471	30.76	49.3	26.54
Creatinine	101	6.60	41.6	17.82	875	57.15	49.8	28.91
Eosinophils	0	0	0.0	0	865	56.50	43.2	23.70
Erythrocytes	570	37.23	56.7	33.16	10	0.65	50.0	20.00
Glucose	14	0.91	50.0	35.71	519	33.90	52.6	27.17
Hematocrit	590	38.54	58.5	33.73	22	1.44	45.5	31.82
Hemoglobin	484	31.61	62.8	37.81	11	0.72	54.5	27.27
Mean globular hemoglobin (HGM)	68	4.44	60.3	27.94	97	6.34	48.5	24.74
International normalized ratio (INR)	0	0	0.0	0	79	5.16	73.4	48.10
Lactate dehydrogenase (LDH)	3	0.20	100	100	813	53.10	91.5	26.69
Lymphocytes	0	0	0.0	0	1,234	80.60	47.7	26.26
Neutrophils	0	0	0.0	0	1,252	81.78	48.2	26.84
Platelets	146	9.54	48.6	28.77	24	1.57	50.0	41.67
Blood oxygen (pO2)	37	2.42	78.4	37.84	59	3.85	61.0	42.37
Potassium	66	4.31	65.2	27.27	82	5.36	61.0	36.59
C-reactive protein (CRP)	0	0	0.0	0	877	57.28	54.5	30.56
RDW-CV	0	0	0.0	0	446	29.13	59.6	35.65
Sodium	94	6.14	68.1	39.36	21	1.37	61.9	38.10
AST	0	0	0.0	0	746	48.73	50.7	27.61
ALT	24	1.57	54.2	20.83	196	12.80	64.8	38.78
Activated partial thromboplastin time (APTT)	9	0.59	55.6	22.22	212	13.85	53.3	32.08
Prothrombin time	1	0.07	0.0	0	147	9.60	69.4	43.54
Troponin I	0	0	0.0	0	1,319	86.15	47.4	25.17
Urea	0	0	0.0	0	414	27.04	64.5	40.58

Abnormal level results were found more frequently in patients with LOSE ≥ 11 days in the analysis of albumin (59.1% below level), CK (55.6% below level) and LDH, which showed values 100% below level but with a low frequency (3). For the patients with LOSE ≥7 days in LDH (91.5% above level), pO2 (78.4% below level) and Albumin (72.7% below level).

### Validation model

A Recall (58%) and Specificity (73%), with a cut-off of 0.26, and a discriminatory capacity (area under the ROC curve [AUC] of 0.702) were observed for the population of patients with a primary diagnosis of AMI, who were discharged alive and for the variables defined in the previous model (VM). According to the data presented in Table 3, there is a decrease in the predictive capacity of the VM compared to the results obtained in the model by Magalhães et al. (13). The VM considered LOSE ≥ 7 days.

**Table 3:** Performance measures comparison.

**Performance measures**	**Magalhães et al. (13) model**	**Validation model (VM)**
Recall	72%	58%
Specificity	75%	73%
Cut-off	0.24	0.26
AUC	0.828	0.702

As shown in Table 4, after testing the VM in the study population, the confidence interval (CI) did not remain the same in relation to the model of Magalhães et al. (13). In addition, differences were also observed concerning clinical variables:

- Total carbon dioxide level in the blood was not tested, since it was not present in the database;
- Chlorine and prothrombin time had the same reference range. However, the range of results was wider;
- The mean platelet volume had the same reference range. However, the range of results was narrower.

**Table 4:** CI values between the model by Magalhães et al. (13) and the validation model (VM) for AMI.

**Magalhães et al**. **(****13****)** **model predictive factors**				**CI of validation model (VM)**
**Variables**	**OR** ^a^	**CI 95%** ^a^	***p*** **-value**	**CI 95%** ^a^
Age group [69, 100]	3.31	1.9–5.28	0	1.92–5.86
Cardiogenic shock	17.78	1.6–134.02	0.019	2–390.41
Diabetes with complications	37.83	4.12–242.00	0.001	5.71–754.53
Cardiac dysrhythmia	2.97	1.2–6.37	0.019	1.18–7.34
Cerebrovascular disease	18.41	2.12–113.07	0.008	2.97–356.32
Pulmonary edema	7.31	1.38–29.61	0.019	1.58–52.32
Respiratory infections	9.32	1.72–38.47	0.01	2.07–69.29
Neutrophils (above level)	2.03	1.19–3.17	0.009	1.92–5.86
pO2 (below level)	1.7	1.38–3.47	0.222	0.71–3.94
pO_2_ (above level)	4.52	1.41–12.01	0.011	1.42–15.15
Prothrombin time (above level)	1.64	1.09–2.67	0.097	0.91–2.92

^a^OR, Odds Ratio; IC, Intervalo de Confiança do OR.

### Recalibration model

Administrative and laboratory variables with *p-*value < 0.28 were included in the logistic regression analysis for the population of patients with a primary diagnosis of AMI who were discharged alive and whose LOSE was ≥11 days. The variables were age, type of AMI, shock, pulmonary edema, acute renal failure, chronic renal failure, cerebrovascular disease, dysrhythmia, diabetes with complications, anemia, respiratory infection, albumin, calcium, chlorine, creatinine, eosinophils, erythrocytes, hematocrit, hemoglobin, INR, LDH, pO2, potassium, C-Reactive protein (CRP), RDW-CV, sodium, ALT, Activated Partial Thromboplastin Time (APTT), prothrombin time, troponin I, urea.

The explanatory variables of the LOSE were adjusted throughout the tests performed and those that were not significant for the model were removed. Thus, episodes with zero days of hospitalization were also removed, to improve the model's recalibration. The age variable was categorized for individuals aged ≥69 years, thus being included in the potentially predictive variables.

Table 5 shows the final variables, resulting from the multiple logistic regression analysis, which are part of the score of the new LOSE model. The variables are age ≥69 years, CRP (above level), troponin I (above level), shock, pulmonary edema, dysrhythmia, diabetes with complications, anemia, and respiratory infection. As shown, all variables have positive coefficients except for troponin I.

**Table 5:** Predictive factors of LOSE ≥11 days for patients with AMI discharged alive.

**Variables**	**Coef**.	**OR^a^**	**CI 95%^a^**	***p-*value**	**Pr (>|*p*|)**
Age ≥69	0.1245	1.48	1.13–1.93	0.004	**
CRP (above level)	0.1826	1.57	1.20–2.06	0.001	**
Troponin I (above level)	−0.8213	0.62	0.44–0.89	0.009	**
Cardiogenic shock	2.0367	22.96	7.67–99.09	0	***
Pulmonary edema	0.8942	4.34	2.45–7.91	0	***
Cardiac dysrhythmia	0.1452	1.54	1.16–2.04	0.003	**
Diabetes without complications	0.1993	1.85	1.22–2.79	0.003	**
Anemia	0.4890	2.31	1.63–3.26	0	***
Respiratory infection	0.9006	3.55	2.46–5.15	0	***

Signif. codes: 0 “^***^”; 0.001 “^**^”.

^a^OR, Odds Ratio; CI, Confidence Interval; Pr (> …) – Significance.

Variables with a positive coefficient indicate that patients who have this variable have a higher risk of LOSE than those who do not. The opposite is true for variables with negative coefficients.

Patients at greatest risk of LOSE are those who present shock on admission, about 23 times more than those who do not have this comorbidity (OR = 22.96; *p* = 0).

Troponin I (above level) was associated with the lowest risk (OR = 0.62; *p* = 0.009). Thus, patients with these values on admission have a lower risk of LOSE ≥11 days. The most significant variables (^***^) are shock, pulmonary edema, anemia, and respiratory infection.

Table 6 shows the comparison of OR values and 95% CI between the model by Magalhães et al. (13) and RM for the study population.

**Table 6:** Comparison of the values obtained for OR and 95% CI between the models.

**Magalhães et al**. **(****13****)** **model predictive factors**				**Recalibration model (RM) predictive factors**			
**Variables**	**OR** ^a^	**CI 95%** ^a^	***p-*** **value**	**Variables**	**OR** ^a^	**CI 95%** ^a^	***p-*** **value**
Age group [69. 100]	3.31	1.9–5.28	0	Age ≥69	1.48	1.13–1.93	0.004
Cardiogenic shock	17.78	1.6–134.02	0.019	Cardiogenic shock	22.96	7.67–99.09	0
Diabetes with complications	37.83	4.12–242.00	0.001	Diabetes with complications	1.85	1.22-2.79	0.003
Cardiac dysrhythmia	2.97	1.2–6.37	0.019	Cardiac dysrhythmia	1.54	1.16–2.04	0.003
Cerebrovascular disease	18.41	2.12–113.07	0.008				
Pulmonary edema	7.31	1.38–29.61	0.019	Pulmonary edema	4.34	2.45–7.91	0
Respiratory infection	9.32	1.72–38.47	0.01	Respiratory infection	3.55	2.46–5.15	0
				Anemia	2.31	1.63–3.26	0.000
Neutrophils (above level)	2.03	1.19–3.17	0.009				
pO2 (below level)	1.7	1.38–3.47	0.222				
pO2 (above level)	4.52	1.41–12.01	0.011				
Prothrombin time (above level)	1.64	1.09–2.67	0.097				
				CRP (above level)	1.57	1.20–2.06	0.001
				Troponin I (above level)	0.62	0.44–0.89	0.009

^a^OR, Odds Ratio; IC, Confidence Interval.

Table 7 compares the performance measures for the population of AMI patients who were discharged alive, comparing Magalhães et al. (13) model, the VM of this study and the RM.

**Table 7:** Comparison of the performance measures obtained in the models.

**Performance measures**	**Magalhães et al. (13) model**	**Validation model (VM)**	**Recalibration model (RM)**
Recall	72%	58%	69%
Specificity	75%	73%	68%
Cut-off	0.24	0.26	0.21
AUC	0.828	0.702	0.745
Hosmer-Lemeshow test	0.995	-	0.692

## Discussion

The goal of this study was to validate a previous predictive model of extended hospital length of stay (13) and recalibrate it for a different population, by analyzing administrative and laboratory data from an NHS Portuguese hospital regarding episodes of patients aged 18 years or older, with a primary diagnosis of AMI, discharged alive.

In comparison to the model developed by Magalhães et al. (13), the VM developed in this study lost predictive capacity, the Recall and Specificity valuesropped, as did the AUC. As a result, the model required recalibration in the population under study. Nine predictive variables of LOSE were obtained during AMI model recalibration, using the statistically significant variables. Of these, the variables age, shock, pulmonary edema, dysrhythmia, and respiratory infection were present in the validation model. All variables, except for troponin I, had a high predictive capacity of LOSE, and all of them were statistically significant.

When the Magalhães et al. (13) model was compared to the RM, age, diabetes with complications, dysrhythmia, pulmonary edema and respiratory infection had a lower OR (lower predictive capacity). The variables age, shock, pulmonary edema, and respiratory infection had lower CIs. It is also possible to verify that the variables in the RM presented a higher degree of significance compared to the model by Magalhães et al. (13).

The analytical variables were those that differed the most between the models, and there was no agreement on any of them. This fact can be explained by the data collected in the different hospitals and by the different calibrated values of the results.

The results pertaining comorbidities obtained from the recalibration model were consistent with the data obtained by Elixhauser et al. (17), who identified diabetes with complications and pulmonary edema as comorbidities that increase not only LOS but also mortality and hospital costs. These authors also highlight cancer and renal failure as predictive factors; however, they were not verified in this model.

Furthermore, Swaminathan et al. (9); Magalhães et al. (13) and Kaul et al. (18) suggest that patients with LOSE had a higher prevalence of heart failure, diabetes, renal failure, cerebrovascular disease, peripheral vascular disease, chronic lung disease, hypertension, respiratory infections, pulmonary edema, metastatic cancer, coagulopathy, shock, and analytical changes.

The age group over 65 years old is frequently mentioned as being predictive of LOSE and even mortality. For instance, Swaminathan et al. (9); Saczynski et al. (19) and Magalhães et al. (13) conducted studies with elderly populations to test their predictive capacity concerning hospitalized patients with cardiac events.

According to the AMI Risk Score (thrombolysis in myocardial infarction, TIMI), risk stratification is important in predicting disease prognosis. This score serves as a tool to predict death and other cardiac ischemic events. It is composed of seven independent variables: age ≥65 years, 3 risk factors for Acute Coronary Syndrome (ACS), anterior coronary artery stenosis, use of acetylsalicylic acid in the last 7 days, ST-segment deviation of 0.5 mm, symptoms of angina/ chest pain in the previous 24 h and presence of an elevated cardiac marker (CK-MB or troponin I) (20). The tested model shares some variables with this predictive model (age and troponin I).

According to the same study, the Hosmer-Lemeshow test (3.56) and the AUC (0.65) for the predictive power of TIMI for unstable angina/ NSTEMI were lower when compared to the AMI RM (Hosmer-Lemeshow test: 0.692 and AUC of 0.745). Thus, the RM for AMI showed better results and a higher discriminatory capacity (AUC) compared to TIMI. However, further studies should be conducted on this evidence, while increasing the sample dimension, allowing the models to evolve to another dimension, namely in the ability to inform not only about LOS and risk of death, but also the decision of the best treatment given the patient's condition (21).

Regarding the analytical variables, CRP, defined as “a marker of acute inflammatory phase response synthesized in the liver” (22), has been associated with the assessment of cardiac risk in intermediate-risk patients (23).

According to the findings in the Potsch et al. (22) study, CRP levels were higher in patients with a primary diagnosis of AMI compared to those who did not have this diagnosis, so CRP values above the level at the time of admission are a useful tool in the identification of patients with more severe chest pain.

Another interesting finding of this study, which corroborated the observed results, is the fact that the measurement of CRP during hospitalization is an important “tool in the prediction of adverse cardiac events during the hospitalization period” (22).

The LOSE value considered is another difference discovered when compared to the previously validated model. LOSE was defined as ≥7 days in the model by Magalhães et al. (13). However, LOSE was found to be 11 days in this population, based on the 75th percentile of the total days of hospitalization (13, 24). Because the difference in the cut-off point may have influenced the results obtained, the model must always be adjusted to the existing population.

This number of days, which define LOSE, was also found in a study by Li et al. (25), on national trends in length of hospital stay for AMI in China, where LOS remains considerably high, with an average of 12 days in 2011.

Regarding the performance measures for the population of patients with AMI who were discharged alive, the results obtained in the RM were worse compared to the validation by Magalhães et al. (13) and improved compared to the VM (expected result, since the model was adjusted to the population). Although the AUC decreased slightly, the Hosmer-Lemeshow test showed the greatest decrease in values.

This study had some limitations, namely the difficulty in obtaining studies developed within the scope of LOSE and predictive factors of LOSE, for patients with AMI and cardiac pathologies and also the inclusion of other patient-related data as vital signs and cardiovascular exams results.

The study also made it possible to identify further research opportunities, such as:

- Inclusion of new variables, as is the case of vital signs and cardiovascular exams;
- Elaboration of a more detailed analysis of other cardiovascular pathologies, since all these pathologies have different characteristics;
- Applying this methodology to chronic cardiovascular diseases and even in the rehabilitation care of these patients.

## Conclusion

Decision-making support models have proven to be useful in health systems, particularly in hospital management, to achieve the goals of reducing hospitalization time, increasing the number of available beds, and reduce waiting lists. Models that predict the length of stay might be especially helpful to achieve these objectives (10, 16, 26).

This study allowed researchers to conclude that a previous model for predicting LOSE on patients diagnosed with AMI could not be generalized. Therefore, it highlights the need to recalibrate the models to new populations, as the models cannot be generalized to different populations without losing their predictive capacity. However, more research is needed to understand whether these models could be generalized in similar populations and hospitals.

## Data availability statement

The datasets presented in this article are not readily available because the data that supports the findings of this study is only available with the permission of the hospital in study. Requests to access the datasets should be directed to teresa.magalhaes@ensp.unl.pt.

## Ethics statement

The studies involving human/animal participants were reviewed and approved by Comissão de Ética do Centro Académico de Medicina de Lisboa (Ref. nr. 9/16).

## Author contributions

JX and TM designed the study and carried out the data collection. JS wrote and revised the paper. TM coordinated the study and revised the paper. FP is a cardiologist doctor and the data donor and also revised the paper. All authors read and approved the final manuscript.
